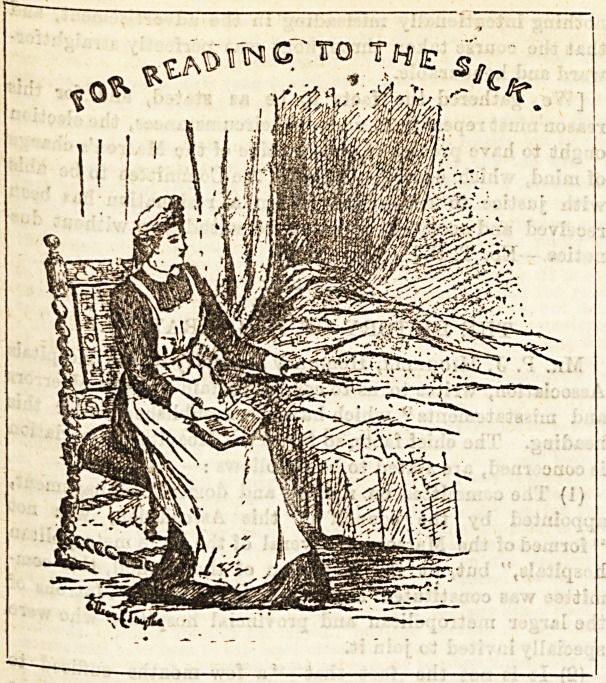# The Hospital Nursing Supplement

**Published:** 1892-07-30

**Authors:** 


### "The Hospital" Nursing Mirror.

Being the Extra Nursing Supplement of "The Hospital" Newspaper.

Contributions for this Supplement should be addressed to the Editor, The Hospital, 140, Strand, London, W.C., and should have the word "Nursing" plainly written in left-hand top corner of the envelope.

### En Passant.

REGISTRATION OF NURSES.—The hearing of the petitions for and against the application to grant a Royal Charter to the R.B.N.A., which was provisionally fixed to come on at the end of this month, has been postponed by the Lord President of the Privy Council until later in the year.

SOME OF THE EFFECTS OF CLEANING.—The entire closing of a hospital, such as University, for six weeks during its cleaning operations is certainly an unmitigated evil to the poor of the neighbourhood. They are heard dolefully remarking, "University closed and the Royal Free full, and cleaning going on there, too!" Yes, it's hard lines on the poor who are really ill, and must wait in their miserable homes bearing their burdens without the alleviation of skilled nursing and other comforts. But to the ministering women our thoughts can go with pleasure, for it is indeed satisfactory to know that University nurses are all off for six weeks' holiday, and sincerely do we wish for them and for all summer wanderers "a good time" and a safe return.

KELSEY v. McLELLAND.—A case was heard on Thursday, the 21st, at the Birmingham County Court, in which plaintiff and defendant were connected with the nursing world. Harriet Belsey, of Prestwich, Manchester, brought an action against May Jane McLelland, Matron of the Worcester Infirmary, to recover £500 damages for assault and libel. Defendant paid 10s. into court in satisfaction of the damages for assault, if any had been committed. Nurse Belsey had signed an agreement with Miss Barnfather, of the Medical and Surgical Home for Patients and Training Nurses, Manchester, for three years, the first of which was to be spent in training at Worcester Infirmary, and the remaining two in private nursing. The assault complained of was that Miss McLelland painted Nurse Belsey's ulcerated throat against her will with the assistance of Nurse Hughes. When the Matron put the depresser into the nurse's mouth she was pushed away, and so she asked Kelsey what she meant, adding, "You are making a fool of yourself." She then tried a second time, but plaintiff asked her to allow her to go home, to which request the Matron replied in language more forcible than elegant. The painting of the throat was then achieved with Nurse Hughes' assistance, and some gargle was given plaintiff to relieve her throat. The alleged libel was contained in a letter written by defendant to Miss Barnfield, in which she said she was sorry that "Belsey has turned out so stupid, but I never met any girl of her years so childish and stupid." The jury naturally enough found that there was no assault and no libel, and judgment was given for the defendant, the Judge remarking that he thoroughly agreed with the verdict. There is nothing very much to gain from this case, excepting that it makes one reflect on the want of dignity which lets people drag little private squabbles into the courts, and before the light of a critical public; it also acts as an additional warning to would-be nurses to remember that perfect health is essential to a nurse's success, for it appeared in the evidence that the plaintiff had previously left a Manchester hospital owing to a break-down of health, and it is also good to remember that vulgar epithets are always unpleasant, likewise unnecessary, and are not permissible whatever the provocation. Vociferous language is, alas! one of our latter developments with which we would willingly dispense.

ROYAL BRITISH NURSES' ASSOCIATION.—The annual meeting of this association took place at Brighton, at the Royal Pavilion, on July 21st. A special train conveyed friends and members from town, and amongst those present were Dr. and Mrs. Bedford Fenwick, Miss C. J. Wood, Miss Cartwright (of the Gordon House Home Hospital), Dr. Bezley Thorne (Hon. Secretary), and others; Dr. Hollis presided over the meeting. The number of nurses now on the roll are numbered at 2,818; the year's expenditure was £734, out of an income of £980. After the meeting, luncheon was served in the banquetting-room, under the presidency of the Mayor of Brighton, and in the afternoon many of the visitors inspected the Sussex County Hospital and the Brighton Home of Rest for Nurses. The Council, with the addition of Dr. Hollis, was re-elected.

DEATH AT LAMBETH.—An inquest has been held on the body of Caroline Foster, aged seventy-eight, a widow, lately living in Coral Street, Lambeth, who died on the 20th instant, and who was lately an inmate of Lambeth Infirmary. Caroline Payne, niece of the deceased, gave notice at the infirmary on Sunday, May 8th, that if the aunt kept well till the Thursday she should remove her to her home. When Caroline Payne went to bring away Mrs. Foster, the old lady (who had an impediment in her speech) was pushed into the bathroom by the nurse in a naked state, screaming all the time, on which the nurse said, "She's always screaming;" and when they tried to dress her, her screams were so terrible that they were unable to do so. The niece obtained a discharge card from Dr. Lloyd, who remarked that the deceased was very old and infirm, and it was silly to remove her. The old lady was unable to walk, and so was helped into a cab and taken home, when it was found that she was much bruised and swollen, and on subsequent examination Dr. H. E. Richardson, who was called in, discovered that her leg was fractured at the lower end of the femur, near the kneecap. This, it appeared, was the result of a severe fall that deceased had had in the ward, which had caused her much pain, and when picked up she had cried bitterly, yet no word of this accident was told the niece, and the nurse, Elizabeth Watson, stated in the evidence that she had seen no bruises on deceased. Anna Howe, head nurse, stated that owing to Mrs. Foster's complaining of pain in her knee she called Dr. Lloyd's attention to it and had it dressed, but owing to the swollen state of the leg the fracture was not discovered. The old woman, however, sank and died on the 20th inst. The Coroner stated that if the deceased had not been removed, in all probability Dr. Lloyd would have found out the extent of her injuries, and the jury's verdict was as follows: "The jury are agreed that the deceased met her death by accidentally falling down in the ward of the Lambeth Infirmary. They also think that the officials should be severely censured for not attending to her sooner, and also for not informing her niece, Caroline Payne, of her fall." Can we wonder that the readers of our daily papers shudder at such cases as these, and pray that fate may never bring them to a pauper death-bed, where, perhaps, we may be some day be described as "always screaming," and no one will discover that our brittle old bones are fractured, and that our screams are those of pain too hard to be endured?

### Ventilation, Disinfection, and Diet.

By P. Caldwell Smith, M.D.

XVI.—DIET AND DIETARIES—(*concluded from p. cxiv*).

Barley — Maize — Rice — Potatoes — Leguminous Foods — Accessory Foods—General Remarks as to Diet—Diet for Soldiers, for Infants, and for Young Children — Diet in Certain Diseases.

Barley was at one time used largely as a food in the shape of barley meal, but now it is only used in the form of barley water, and in some forms of vegetable soups, the greater amount being converted into malt for the production of whisky. Barley is a very nutritious food, and is a very cheap one as compared with flour and oatmeal. One pennyworth of flour will contain 6·3 oz. of nutritive matter; one pennyworth of oatmeal, 5·7 oz.; while one pennyworth of barley will contain as much as 13 oz. Barley water should be used much more largely as an invalid diet. Maize or Indian corn, from which corn flour is made, is a very good form of food, one pennyworth of this maize containing 12½ oz. of real nutriment. Of course, corn flour does not contain the same amount of nutriment as maize, but it is a much more palatable food, although more expensive.

Rice is a food which is very largely used in some parts of the world—in India it is the staple food. It is not used much in this country, except as an addition to other foods, as it contains so little nitrogenous matters and fat.

Potatoes are mainly composed of starch, but with the addition of fat, as in butter or bacon, they form a fairly nourishing food. This is in Ireland a diet much used, and where the pig is reared on the premises it is a very cheap diet.

It has been calculated that for an adult half a pound of bacon and 5 lbs. of potatoes would be necessary for moderate work, or even hard work. It would contain 8 oz. albuminoids, 3 oz. fat, and 16 of carbo-hydrates.

The various leguminous foods, such as peas, beans, and lentils, contain a nitrogenous substance called legumen, which is a vegetable albumen. They are all very nutritious, but should be well cooked if the greatest benefit is to be derived from them. It has been found that if badly cooked only about 60 per cent. are absorbed, while if well cooked, nearly 92 per cent. are absorbed. The one thing that all these leguminous foods require is fat, hence we take bacon with beans, and butter with peas.

We are also in the habit of using a large number of what are called succulent vegetables, as cabbages, carrots, turnips, parsnips, marrow, asparagus, &c. These are useful, as they contain vegetable acids, such as citric and tartaric, which play an important part in the economy. Without them we are very liable to become victims to various skin eruptions, and this is well seen in the case of sailors, who if deprived of these for some time, suffer from scurvy.

We now come to consider the various accessory foods, as tea, coffee, cocoa. The first two are mainly used as drinks, which in some way or other impart some energy to the nervous system, while cocoa is more of a food, as it contains a large proportion of nutritious substances. The tea drinking habit has increased very much within the last thirty or forty years, and among the poorer working classes it is used at nearly every meal, to the ruin of their digestion and nervous system.

Tea is of very complex composition, containing a large proportion of tannin and woody fibre, besides nitrogenous matters, and a small quantity of fat. The essential constituent is theine, and the quantity of this in tea varies from 1 per cent. to 3 per cent. A cup containing half a pint of tea wi'l contain about 2½ grains of theine and 7 grains of tannin. Indian tea contains a larger percentage of tannin than China, but the flavour, which depends on the essential oils, s somewhat different.

Coffee is not so much used as it was once. To get good coffee the berry should be roasted and ground at once. Most of the coffee bought in shops contains a certain proportion of chicory, one analysed lately containing as much as 95 per cent. The alkaloid contained in coffee is called caffein, and is used as a drug by physicians.

Cocoa is got from the roasted seeds of a tree which is cultivated in Brazil, Peru, East Indies, Mauritius, Madagascar, &c. The main ingredients in cocoa are theobromine and cocoa butter, which is a kind of fat, as much as 46 per cent. of this being present in pure cocoa. The most of the cocoas sold as soluble cocoas, homœopathic cocoas, &c., are mixtures of cocoa with starch and sugar in varying proportions.

Alcohol, although included by some in the list of accessory foods, should be regarded at least by us as a medicine, and consequently I should not enter on a discussion as to its merits as an article of diet.

We have seen that for the proper nutrition of the body we must have nitrogenous foods, fats, carbo-hydrates or starches, and salts or mineral matters in a certain proportion if we are to keep in perfect health. The amount of these I have already stated, and I have also called your attention to the fact that all diets must be mixed, and have a certain amount of variety. The regulation diet of an English soldier may be given as an example of what may be called a scientific diet. It consists of 12 oz. of meat, one-fifth of which is bone, 24 oz. of bread, 16 oz. of potatoes, 3 oz. other vegetables, 3¼ oz. milk, 1·3 oz. sugar, with salt, tea, and coffee. I would be inclined to substitute for tea or coffee cocoa, which contains, as stated, some fat, and some of the leguminous foods, as peas or lentils. Lentil soup, for example, is one of the most nourishing soups we have, and if made with milk, is an absolutely perfect food.

For infants the proper diet is, of course, milk, and this is true of other animals besides man. Up to the end of the eighth month no child should get anything else but its mother's milk. The reason for this being fixed at the eighth month is that up to that time the saliva of the child does not contain so much of the ferment which acts on starch as it does when the teeth are through. At that time some of the farinaceous foods, baked flour, &c., should be given, and as the child grows older a larger proportion of farinaceous food may be given. If a mother is unable to nurse her child, then cow's milk should be given, and not the milk of one cow, but the mixed milk of all the cows in the cowshed, as by doing this a good average milk is got. To this cow's milk water has to be added, as already stated, and sugar ¼ oz. to the pint, or better, sugar of milk 1 oz. to ⅔ pint.

For young children a large amount of starchy food is necessary. At the Orphan Asylum at Munich the following is the diet given:—

Breakfast.—9 oz. milk, 1·4 oz. bread.

Mid-day Meal.—A vegetable dish composed of 1·8 oz. cabbage, 6 oz. flour, ·4 lard, with onions, 6 oz. beef, [including bone, mashed potatoes made of 7 oz. potatoes, 5oz. flour, 3 oz. lard, and a couple of onions; also 2·8 oz. bread.

Afternoon.—2 8 oz. bread.

Evening Meal.—Bread 2·8 oz., beer half-pint, 9·9oz. potatoes, cut in slices, with 4·4 oz. lard.

In nearly all diseases the quantity of food taken, the intervals between meals, and the quality of the food has to be regulated, but one maxim to remember is that the circumstances and previous habits of the patient have always to be considered before altering the diet in any way. For instance, take a man who had always been in the habit of taking porridge, bacon, cheese, &c., as staple articles of diet. If he is struck down with any disease not inflammatory, that is, in which the temperature does not rise above the normal, it would never do to give him veal soups, calf's-foot jelly, beef tea, &c., with bread, biscuits, or strong meats. There should be, as Pavy puts it, no great deviation from what is natural.

### Probationers.

A word which is constantly in use not unfrequently loses something of its real meaning through its familiarity, and this term of "probationer" is a good illustration of the fact.

We are so used to hearing a certain section of the army of nurses described as "pros" by their companions, and as probationers by their superior officers, that we seldom attach any meaning to the word, probably considering it only a kind of distinctive title. Moreover, how many of these young nurses themselves continue to feel, say after two or three months in hospital, that they are still on trial? When custom has familiarised them with the routine, and their first inexperienced blundering has ceased, they naturally lose both the appearance and the sensations of new probationers, and feel quite competent to criticize the peculiarities of the last new comers.

At some hospitals one preliminary month is given, during which it is considered that the aspirant will have an opportunity of deciding whether or not a nurse's life is her vocation, and the experienced women under whom she works have the responsibility, on the other hand, of judging what promise she gives of fitness for training.

It is almost impossible for those who have never gone through such work to estimate the many difficulties which beset a matron and a sister in these matters. They have to decide a question which may be of vital importance to another woman, which may affect her career, not only for the moment, but for life. They face a risk of condemning one who has noble capabilities, but who is so slow at adapting herself to new surroundings that she gives no outward sign of her natural gifts; and they are still more likely to accept as a valuable acquisition the far shallower girl to whom novelty acts as a spur, and whose seeming ability will evaporate with habitude.

Some people can do very well for a month, and others take that time to learn how best to set themselves to the tasks which they have taken up with a seriousness which is little short of a solemn personal dedication to the service of the sick.

To some ward sisters is given, by Nature, great insight into character, and this gift, when matured by experience, can be utilised for the decided benefit of probationer and institution.

Although failure proves detrimental to the candidate, it is eventually more disastrous to patients and fellow nurses when an utterly incompetent person, having safely got through the initial month, is settled in their midst for a period of two, three, or four years, during which everybody has to make the best of her (bad as that best may be) unless she makes a false step, which cannot be condoned, which ends her career in that special institution. Of course, some hospitals hold a higher standard of health, manners, and "tone" generally, and from these a vast number of probationers are weeded out during the first year of trial, but a great many naturally refrain from this plan, giving dismissals on serious grounds only, and considering "incompetency, after fair trial," does not come under this head.

Now that fashion, amongst other motives, keeps the probationer-ranks so well recruited. we may hope to find quality as well as quantity efficiently supplied. We want to see the best kind of woman receiving the highest possible training; the lady to whom the old-fashioned appellation of gentlewoman belongs, not the fine lady, who says service is menial, and prefers putting on a bandage (probably badly!) to bathing and cleansing the unfortunate creature, who needs the two latter quite as much as the former. Glad as we have been to welcome a better-educated class of workers into hospitals, we are still constrained to own that the station from which superior housemaids and head servants in general are drawn is still the most practicable. These young women are usually patient, observant, and quiet, as well as personally neat. Having given up comfortable and well-paid domestic service to get this coveted "training," they have generally done so from genuine affection for sick-nursing, and they are correspondingly anxious to make the most of their opportunities, aud to do well by their patients.

Of the earnest, highly-educated woman, who leaves a luxurious home, where her duties can be fully carried out by her sisters, to come into the field of real work, we see pleasant examples very often; and her utility is not confined to the ward, for she is able, by means of her personal influence, and also through her prosperous relations, to do a great deal towards the after care of the patients, and probably, too, she takes many of the opportunities which offer for befriending those nurses less happily circumstanced than herself.

We were very much struck by the remarks of an experienced and successful matron, who was graphically describing her first probationer days, which had begun in the early times of "trained nurses." After giving an amusing sketch of the food and mode of serving it, which was thought suitable at that period, she proceeded to speak of her first night's experience in a hospital: "It was before nursing homes existed, at least, they were little known or thought of, and few people imagined that the day would come when cosy little separate bed-rooms would be the rule, and not the exception. I found myself in a long dormitory divided off by curtains, which shielded us from our neighbours' eyes; but, oh! how I longed for an effectual protection for my ears. It seemed to me that the half-hour which elapsed before the gas was lowered would never end. I was not unsociable by any means, and in a few weeks I had laid the foundations for several friendships which will only end with life itself; but sociability was not the prevailing atmosphere in the dormitory that first night. It was, to me, whose home life had been a calmly happy one, such an awful revelation of what might have been my lot, that even now, after all these years, I can feel the deadly depression which fell on my spirit as I listened to the remarks of my room-mates. They were all fairly new probationers, and their interests in their fresh life were interspersed with past trials. With hardly an exception they spoke with discontent of their homes and their parents. With one girl a step-mother was to blame for everything, although some natural rivalry in housekeeping was the only obvious source of disagreement; another was 'put upon' by her sister; a third had transgressed her father's commands in the matter of a lover; a fourth was 'sick of doing nothing at home,' and many others were misunderstood by their nearest relations. A very useful word this, 'misunderstood,' by the way, it justifies many a girl to her own conscience, when she has first failed in her natural duties, and then neglected them to take up more picturesque ones in which she anticipates that she 'will be appreciated.' A little more experience of my fellow pros. showed me that my first view was exaggerated, and the recruits were not all discontented with their homes, but all through my nursing career I have always found a certain proportion of probationers who have taken up the profession 'to get a change.'"

It is the women who have been useful and unselfish in their home lives who, naturally enough, prove the greatest acquisitions in hospitals; they are in sympathy with others and forgetful of themselves, and are far oftener possessed of the "healthy mind in a healthy body" than those damsels who indulge in unwholesome mental dissection, and overvalue the personality which they imagine the world in general under estimates.

If probationers begin their course of training with a firm resolve to study the patient's comfort before their own, and to be absolutely loyal to their hospital, feeling that they have a share in raising and maintaining its reputation, they will find rules easy to keep and matrons and sisters quick to appreciate their endeavours.

### Our American Sisters.

We English nurses are apt to be very narrow in our views. We each of us think, perhaps naturally, that our own particular training school is the centre of the universe, and we are slow to acknowledge the possibility of equal excellence n any other.

No doubt this feeling about nursing is a part of the system which Lord Sandhurst referred to at a meeting the other day, when he said the Lords' Committee "would be extremely sorry to see individual hospitals interfered with, for the reason that the rivalry they promoted tended to their administrative efficiency."

As such efficiency must be the result of progress, so perfection in nursing can be obtained only by friendly competition, and by the emulation of all that is best in other countries as well as our own. We cannot all of us aspire to wander in strange lands, and we must perforce content ourselves with hearing, instead of seeing, how other nations nurse their sick.

Liberal, go-a-head America, is certain to have something to teach us, and we find that nurses on that great continent are paid at a far higher rate than they receive here, and therefore, although living and clothing are much more expensive, more money is certainly made by our American sisters. But do not let any English nurse be carried away by this idea, and imagine for a moment that she could make a fortune if she were to emigrate to America. On the contrary, an experienced New York matron told us the other day that, although the number of trained nurses in her country did not yet exceed, or possibly entirely supply the demand, still there were so many probationers in training now, that no English nurses would gain anything by establishing themselves in American cities unless they went there by special request or favoured by a ready-made connection amongst old friends or relations.

Nurses' residential clubs are in no more forward state over there than they are here; in fact we incline, from what we hear, to believe they are practically undeveloped. Yet we may rest assured that when they become a necessity American citizens will be well to the fore, and will teach us a lesson that will be useful to us. Already they are ahead of us in nurses' homes, for whereas a "sitting-room" is shared by scores of nurses even in our best arranged English hospitals, there is one establishment in New York which provides a sitting-room to every two nurses! this ideal arrangement being similar to the plan carried out at the "Alexandra House" students' paradise at Kensington.

The question of ward-maids, as we call them, chambermaids as they call them, has also received considerable attention, and the nurses in America are absolved from many mechanical and heavy tasks, and so left freer to cultivate the higher parts of their calling. It is a pity such hospitals as still allow their probationers to do so much kitchen work do not copy the practice of some of the American matrons. At the National Hospital in Queen Square, where the cases are all, in nurses' parlance, "heavy" ones, the young probationers have so much of the wearisome washing-up and cleaning to do, that they can have but limited strength and spirit left in them to devote to the care and legitimate nursing of a class of patients who especially appeal to the sympathetic instincts of the true nurse.

Whether from an American or an English standpoint, we may safely declare that those gentle and patient women who voluntarily take up nursing in this especially trying department of the work ought to be spared all exhausting manual labour, for assuredly, first the learning, and afterwards the application of the popular "massage" treatment, must give quite sufficient muscular exercise in the course of the day's routine.

To return to our American sisters, we learn from them that patients in their country are not accustomed to as many meals as we give ours. The eight o'clock suppers, which most English hospitals provide, are unknown in their dietary; nor do the nurses either receive or desire any refreshment after the six o'clock supper, which, by the way, is not preceded by afternoon tea. In fact, except as regards the consumption of iced-water, our own countrywomen seem to carry off the palm as "thirsty souls." Tea, coffee, or milk once, or at the most twice a day seem the only drinks there. But here, as one of our New York friends remarked, "even at a temperance hotel, nobody takes plain water as we used to do, everybody has some different effervescing or medicinal beverage."

In yet another point do the New York matrons set us a good example, they arrange for their staff of day nurses to quit work reasonably early in the evenings, leaving at least an hour and a half for study or friendly intercourse before bedtime; this recreation being exclusive of time off duty during the day, when outdoor exercise is encouraged.

So far there seems to us plenty of scope for friendly rivalry with our trained sisters in the New World, who also cheerfully prove their willingness to follow on in good lines already laid down, by the enthusiastic rapidity with which they have received and adopted the scheme of our National Pension Fund for Nurses, which owes so much of its stability to the munificence of an American gentleman.

*The Trained Nurse*—the nursing paper in New York—gives many pages in the July number to the consideration of our successful R.N.P.F., and we shall expect shortly to witness an amicable competition between the two "Funds."

### Nurses' Bookshelf.

### THE WIFE AND MOTHER.*

This small Manual, by Dr. Westland, contains a great deal of information and some practical and wholesome advice to married women. The diet of the mother and her infant are exhaustively dealt with, but we cannot agree with Dr. Westland's suggestion that, "if the baby is making satisfactory progress a little farinaceous food may be given" after the third month. Most doctors are agreed that the "satisfactory progress" would be most likely arrested by any nourishment beyond milk being given to a normally healthy child under six months of age. It is too much the fashion to regard these so-called farinaceous foods as a substitute for milk; this they are not, but they in some cases may act as vehicles for its administrations. Nothing is better than good milk if a child seems to do well on it. The remarks on the clothing of older children are excellent, and so are some of the simple remedies suggested for small ailments, but we cannot praise the heroic treatment proposed under such circumstances as the presence of foreign bodies in the ear or nose. In the former the energetic use of a syringe by unexperienced hands frequently results in a disastrous impacting of the intruding substance. Whilst to find that awful weapon, a crooked hair-pin, recommended as a desirable instrument to introduce into the delicate nostril of a frightened child, makes the reader shudder. With all due deference to Dr. Westland's opinion, we should certainly prefer to seek surgical aid before, and not after, such dangerous experiments are indulged in.

* "The Wife and Mother," by Albert Westland, M.D. (Published by C. Griffin and Co., Strand. Price 5s.)

### MISTS.

In sickness we often raise mists between ourselves and God. We do not mean the thick darkness which hides Him from our eyes altogether, and in which we grope like blind men, but the little fumes of anger and bitterness and jealousy, in fact every form of selfishness, which, like a fog, distorts near objects and makes distant ones almost invisible. Still in our clearer moments we know that it is our own faults and not a lack of God's mercy which ails us. We long so strongly for what is not expedient perhaps, and pine because the very nature of our illness, or their own urgent cases of business, keep our relations and friends from our side. But if we have wise hearts we will not allow these thoughts to veil the eye of heaven from our sight. Man in his weakness needs a stronger stay on which to lean than he can get from the holiest and dearest of his fellow men, so our Father gently untwines the hands which cling to these feeble supports and Himself leads us through the gloom which Christ has trodden before. Fretting and fussing because we must lie still, or cannot have pleasanter food than that which our wise doctors and nurses prescribe, is an easy way to get a mist about us. Try to think of our friends and attendants with kindness and consideration, and do not let us imagine that everybody is against us. We shall soon have a clear vision if we believe and keep in our hearts the truth that God overrules all the circumstances of our lives, that He has put us in a place where, though our bodies suffer, our hearts and minds are moulding to His will.

The light of heavenly love transforms common meals and homely duties into sacraments of His presence, and as He draws near us in love, so we will draw nearer to Him, also fixing our gaze beyond the clouds which now hinder our sight. For the Sun of Righteousness clears away fumes and passions from our hearts, just as the great orb of day disperses earthly fogs and vapours in a marshy land. By keeping our eyes steadily fixed on that Light our troubles and sorrows will pass away, and our sick bed turn into a couch of rest. In this Light we can see things in their proper proportions without exaggerating our own trials or the shortcomings of other people. We are content to rest in the promise of a blessed future, though our path to glory is "darkened and unblest by many a cloud."

Open our eyes, Thou sun of life and gladness,

That we may see that glorious world of Thine!

It shines for us in vain while drooping sadness

Enfolds us here like mist. Come Power benign

Touch our chilled hearts . . . .

Nor by the wayside ruins let us mourn,

Who have the eternal towers for our appointed bourne.

### The Nurses' Journal Club.

Johns Hopkins Hospital, Baltimore, U.S.

Annual Report for the Year Ending May 23rd, 1892.

The first meeting of the Journal Club was held November 16th, at eight p.m. The resignation of the Chairman of the previous year (Miss Macdowell) having been received, Miss Nutting was elected to fill her place. Miss Williams was elected Secretary.

The subsequent meetings of the Journal Club have followed in their regular order—that is, every fortnight—except when unavoidably postponed. During the winter there have been eleven meetings. We have had three original papers—one by Miss Macdonnell on "Symptomatology"; another by Miss Graham on the "Preparation and Use of Salt Solution"; and a third—a carefully-prepared article by Miss Dock—on Dr. Welch's lectures on "Croupous Pneumonia."

In addition to the original articles we have had an unusual number of interesting contributions, which have been selected from the various medical periodicals, notably one read by Miss Barnard on the "Value of the Study of Medicine," an address given at McGill University, Montreal, by Dr. Burke, of London, Ontario; and a very interesting paper read by Miss Laing on the "Symptomatology of Insanity," by Dr. Wood, Philadelphia.

Others worthy of note were those selected by Miss Macdonnell on the "Methods of Zadig in Medicine," an inaugural address given before the Medical Society of London; by Miss Chamberlain on "Missionary Work in East Central Africa"; by Miss Rutherford on a "British View of American Surgery," by Dr. Rutherford Morison.

The following is a detailed report of the work of the meetings for the year ending May 23rd, 1892:—

December 14th.—"The Value of the Study of Medicine," Dr. Burke, London, Ontario, read by Miss Barnard; "Wound Infection, and the Principles Underlying it," by Dr. Welch, read by Miss Dock.

January 11th.—"Salt Solution, its Preparation and Use," Miss Graham, original; "The Tonsils as Gatekeepers," editorial, *New York Medical Journal*, read by Miss Richart; "Hospital Poems," W. H. Henly, read by Miss Nevins.

January 25th.—"Symptomatology," Miss Macdonnell, original; "The Method of Zadig in Medicine," address to the Medical Society of London, read by Miss Macdowell; "The Symptomatology of Insanity," by Dr. Wood, Philadelphia, read by Miss Laing.

February 8th.—"Why Women do not Succeed in Business," read by Miss Nevins; "Medical Gynecology," read by Mrs. Emory; an address to the Club on the "Aims and Possibilities of a Journal Club," read by Miss Hall.

February 20th.—"Two Methods of Treating Diphtheria," read by Miss Pope; two short articles on Sir Morell Mackenzie and the question of Paying Hospital Nursing Pupils, by Dr. Roosevelt, read by Miss Jack; "Selected Poems," Miss Sharp.

March 14th.—"The Treatment of a Cold," Dr. Henry Schroeder, read by Miss McKechnie; "Capital Punishment," read by Miss Dock.

March 28th.—"The Water Bed," and "Pneumonia with Meningeal Symptoms," read by Miss Rudolph; "Asepsis and Antisepsis," an address by Dr. Kelly, read by Miss Toulmin; "The Progress of Hygiene," article published in *The Trained Nurse*, read by Miss Smith.

April 11th.—"Medical Missionary Work in Eastern Central Africa," read by Miss Chamberlain; "A British View of American Surgery," by Dr. Rutherford Morison, read by Miss Rutherford.

April 25th.—Address on opening the Institute of Hygiene, University of Pennsylvania, Dr. Weir-Mitchell, read by

Miss Hemming; "The Infectiousness, Contagiousness, and Heredity of Leprosy," medical and surgical record, read by Miss Townsend; and a paper on "Croupous Pneumonia," as presented in Dr. Welch's lectures to the post-graduates, by Miss Dock.

May 9th.—The meeting of the club on this evening was postponed in order that several of the members might attend the final meeting of the Historical Club of the hospital.

May 23rd.—With the meeting of this date the Journal Club closed for the year. The papers which had been prepared for this evening were omitted, and the time was taken up with a short address by Father Huntington, of New York, and the reading of the Chairman's annual report.

### Everybody's Opinion.

*[Correspondence on all subjects is invited, but we cannot in any way be responsible for the opinions expressed by our corresponden s. No communications can be entertained if the name and address of the correspondent is not given, or unless one side of th3 paper only be written on.]*

### *RE* LONGTON COTTAGE HOSPITAL.

"Medical Correspondent" writes again as follows:—I fear that Mr. Thomas Blair, for his own ends, assumes an engaging innocence and an ignorance of right and wrong which he does not really possess. Possibly it may help him to a right understanding of the case if I point out that the conditions are exactly those of a race. Fifty-four runners enter for it, well knowing that only one can win the prize. Each pays so much as an entrance fee, this in the present instance being represented by the cost of postage, testimonials and photographs. On the day fixed, the competitors come, but are coolly told by the authorities that they must not start, as the prize has already been given to the last year's winner. The entrants, therefore, get nothing whatever in exchange for their money, and do not even have their entrance fees returned. Now does Mr. Blair seriously expect us to believe that he thinks this right? Again, in making would-be facetious remarks about a lady changing her mind, he is simply trifling with the subject. Are we to understand that she voluntarily resigned and then begged to be reinstated without any cause whatever? The whole affair is at present a mystery; and as you, sir, suggest, it would doubtless help us to a truer view of the case if we were allowed to know the details which led up to this extraordinary action of the managers.

Mr. Thomas Blair also writes: I have only just seen your remarks in last Saturday's Hospital upon my reply to a "Medical Correspondent," and regret that you should have been so grossly misinformed as to the circumstances of the case; or, if that be not so, that you should have made any comment without first ascertaining the facts. I am perfectly aware of the custom in advertising which you mention, and also that such an intimation ought to be given where the appointment is not actually an open one. In this case, however, it was in every sense of the term open, as the following facts ought to show: The Matron, for reasons of her own, tendered her resignation to the Directors at their ordinary monthly meeting. This they had no other course but to accept; and, there being no ground whatever for supposing that such resignation would be withdrawn, advertisements were inserted in the usual way, applications to be sent in by July 2nd. But, on the evening immediately preceding this date, a letter was received asking permission to withdraw the resignation, no intimation of such intention having been previously given. Now, sir, these being the simple facts, which I regret you did not know sooner, I quite believe that you will see, and be prepared to admit, that there could be nothing intentionally misleading in the advertisement, and that the course taken throughout was perfectly straightforward and honourable.

[We gathered the facts to be as stated, and for this reason must repeat that, under the circumstances, the election ought to have proceeded, irrespective of the Matron's change of mind, which came too late for the Committee to be able with justice to act upon. When a resignation has been received and accepted, it cannot be withdrawn without due notice.—Ed. T. H.]

### THE HISTORY OF REGISTRATION.

Mr. P. J. Michelli, Honorary Secretary of the Hospitals Association, writes to us to correct certain "material errors and misstatements" which have been published under this heading. The chief facts, so far as the Hospitals Association is concerned, are stated to be as follows:—

(1) The committee on nursing and domestic management, appointed by the council of this Association, was not "formed of the Matrons of several of the large metropolitan hospitals," but, at the invitation of the council, this committee was constituted and consisted of certain Matrons of the larger metropolitan and provincial hospitals who were specially invited to join it.

(2) It is not the fact that "a few months sufficed to prove to this committee that an attempt to organise nurses under the control of laymen would be fraught with the gravest difficulties, if not danger," and the minutes of the committee and council contained no such resolution or expression of opinion.

(3) The council "did not issue in July, 1887, a circular offering to register nurses under the rules" as published. These rules were drawn up by the committee, of which Mrs. Bedford-Fenwick (née Manson) was chairman, and Miss Catherine J. Wood, honorary secretary. They were put into type but were never circulated as stated.

(4) The leaders of the nursing profession did not decline to have anything to do with "this scheme," because it was never submitted to them for their approval or consideration.

(5) It is not a matter of history that they (the leaders of the nursing profession) "retired in a body from the Hospitals Association." What happened was this: The majority of the matrons on the committee retired from it because they disapproved of the way in which its business was conducted, and they have remained in sympathy with the Association ever since. A minority of the committee, when the majority withdrew from it, including Miss Manson and Miss Wood, who were, of course, responsible for the conduct of its business, alone retired from the Association and started the British Nurses' Association.

(6) The National Pension Fund for Nurses is not a commercial undertaking, but is under honorary management, and all the income and property of the society must be applied solely towards the promotion of its objects, and no portion of its funds can be paid directly or indirectly to the members of the Pension Fund, which is conducted entirely and wholly for the benefit of the annuitants and policyholders, who can never become members.

(7) Dr. Steele did not sign the memorandum of association of the Pension Fund, and its articles provide that a register for trained certificated nurses may be provided at the instigation of the representatives of the nurse training schools. Not only has Mr. Burdett not "publicly stated that these powers will be utilised if the opportunity occurred," but, on the contrary, he has declared that such a register could only be undertaken at the unanimous request of the nurse training schools, a condition which is never likely to be fulfilled. I have ascertained that the council of the fund have no wish to exercise these powers under any circumstances.

### NURSING IN ASYLUMS.

"Asylum Nurse" writes: I would like to write a few lines in reference to the article recorded in last week's Hospital on "Asylums of the World." It is a very customary thing for asylum nurses and attendants to be unfairly judged, and held up as bad examples. There are, of course, exceptions in asylums, as well as in hospitals, and while bad exceptions are often met with in hospitals, you will admit that good ones are to be met with in asylums. I know of no institution in the world where the nurses are perfect, or do their duty any too well. I have learned that good and bad will always live together. I think there should be a little more kindness shown for those who nurse the insane. At present there is very little encouragement for us. I don't know who the writer of the article in question may be, but I think he would do well to have a little more asylum experience, for then he would be able to judge for himself if insane nursing is the easiest of nursing. It is one thing to walk through an asylum when the work is done, and the most troublesome patients are sent out under care of special nurses, but it is a different thing to live in an asylum, and to have every day experience of the patients. I think since the writer of the article in question admits himself that a change has taken place for the better, he might have reserved the late Dr. Browne's quotations, and Dr. Clouston's writings. As it is, we are often obliged to listen to false statements about asylums and their nursing staff. I have had nearly four years' experience in this asylum, and I may add that my experience of asylum nursing has not been a bad one. I did not seek this post because I was a lazy body, or the outcast of some other trade. I have not been a servant in either hotel or lodging-house. I did not seek it because I wanted good food and raiment. I have been accustomed to have those all my life, so I had no occasion whatever to enter an asylum for that purpose. I believe a great many nurses prefer asylum nursing to hospital nursing, as they are not so closely confined, and the work is more interesting and very many young people object to the monotony of hospital nursing, but I believe in every nurse doing the work she is most adapted for. It is quite as essential for insane patients to have good nurses, as it is for any other class of patients. I speak not only for myself, but for several of my fellow nurses who have been in this institution for many years, and whom I believe to be highly respectable, and not lacking in educational capacity, I am proud to say this institution is not the receptacle for the lazy rejected outcasts of other trades. I give our superintendent credit for having more respect for the patients and for the old nurses of this institution than to engage a set of lazy men and women. I dare say superintendents are sometimes mistaken in engaging persons, from the fact that employers often give a good character when it is undeserved. When such is the case a doctor is not to be blamed for the faults of other people, and I am quite sure that our superintendent would not hesitate to dismiss any such person if he found out that they were really bad characters and unfit for their occupation. Are we to expect good and respectable nurses and attendants in our asylums when we are publicly condemned by such people as the writer of last week's article? I can only hope that those who read it will not think as badly of us as the writer does. There is much responsibility attached to an asylum nurse, and we frequently run great risk of our lives, how much better it would be then if instead of condemning us, our friends would give us some little encouragement. We are not all as ignorant as the writer thinks, and should know how to appreciate such encouragement.

### TRAINED MALE NURSES.

"Trained Hospital Male Nurse" writes: As a trained hospital nurse of thirteen years' experience, I should like to express my feelings on behalf of male nurses, as I do not consider your article on male nurses and the Hamilton Association, which appeared in your issue of July 2nd, quite fair. In it you say many doctors strongly object to employing male nurses for nursing the sick. That is very true, but how many doctors and patients have been imposed upon by having an ordinary attendant sent to them, instead of a thoroughly-trained hospital nurse, and many are to be had trained in the same school as myself, namely, the Army Medical Staff Corps. I do not allude to men of three years' service; many of these men, I can positively state, have had very little practical experience in a hospital ward, but are notwithstanding classed on discharge as trained hospital nurses, and they obtain employment at an association, to the detriment of those who are really entitled to this calling. Again, you speak of comparing the ordinary man with the highly-trained female nurse. It would appear, according to this, that there are no highly-trained male nurses. Speaking of my own experience, I think many female nurses would be glad of the opportunity of gaining half the knowledge and experience I have had in nursing, both at home and abroad. During the Egyptian Campaign of 1882, at a Mediterranean hospital, I often had upwards of 60 patients under my charge, many of these cases of enteric fever, and only three untrained men from a regiment to assist me, and every third night I was on duty in addition to my daily work. Since leaving the service, on one occasion I had to leave a patient before his recovery, and was relieved of my duties by a so-called trained male nurse, and in the course of our conversation I learnt that the only training this man had in a hospital was three months' assistant orderly at one of our military hospitals. If men like this are sent to patients, it is not to be wondered that medical officers object to them. Trained male nurses are able to endure long hours of watching, and are of great benefit to helpless patients, who require lifting and change of position frequently. A few months ago I went to nurse a patient with rheumatic gout; he was a very tall man, though greatly emaciated from disease. He told me of the two female nurses that had been with him, neither of them could move him without his son's assistance. I could lift him bodily into a chair without any assistance whilst I re-made his bed; which he said had not been re-made for three days; this was verified by his wife, who stated she did not like to interfere with the nurses' duties. The patient was lying in a hollow, owing to the under mattresses being disarranged, and the water bed not properly filled. He had a large bed sore without any dressing on it whatever. The nurse had gone away the day I arrived, and had left no instructions as to the patient's nourishment or medicine; no report on the case was ever furnished to the medical officer; he came and went ignorant of many things he should have been acquainted with. The doctor was very angry at the treatment his patient had received, and but for the patient's wife interceding, would have written to the institution from whence this trained nurse was supplied. I could speak of many other cases of severe negligence and inefficiency on the part of female nurses with the sick, but none of us are infallible, and we are all liable to make mistakes, and all I want acknowledged is that there are good and bad of both sexes. You will probably say I am prejudiced against female nurses, but this I can assure you is not the case. With reference to the scale of fees charged by the Hamilton Association of one and a-half to four guineas, I think the four guinea cases are few. The majority of cases supplied would be nearer two guineas, although I notice the Hanover Nursing Institute for Female Nurses charges three guineas for fever and infectious cases, and I have no doubt many other institutes charge the same. I do not think the rules of the Hamilton Association are very encouraging, as you are required to deposit £3 caution money and to pay an entrance fee of £2, and 15 per cent. is deducted on wages for private cases. Then 10s. a quarter has to be left in the hands of the Association till the amount is £20; this is as a security for the nurses' honesty, and would imply some great necessity for this rule which was not in force some two or three years ago. In some places men on probation get 15s. a-week for six weeks, but the same fee is charged to the patient whether this man be hospital nurse or attendant. Instead of testing the man's abilities by examination and practical ward duty he is sent forthwith to a case, and until some strict measures are taken to ensure patients getting a trained male nurse it would be well for the medical attendant to satisfy himself that the man knows his work before entrusting his patient to the care of an ignorant person.

### The Hamilton Association.

The seventh annual meeting was held at the Westminster Palace Hotel, under the presidency of Sir Joseph Fayrer. Dr. Cox, the secretary, read the report, and stated that the Association was in a better position financially than last, but it is still not self-supporting, and last year had been carried on at a loss, Miss Hamilton having once more come to the rescue. The wages received for the year amounted to £2,541. Since June 1st, 1891, nurses had been on duty at St. George's, Guy's King's College, Westminster, Hampstead Home, Male Lock, and St. Andrew's Hospitals; also at the Seamen's Hospital, Greenwich; the Brompton Hospital for Consumption; the Home Hospital, Fitzroy Square; Chelsea Home Hospital; and the Shaftesbury Union Infirmary. The number of cases which had been completed since May, 1891, was 265, an increase of sixty-two. Sir Joseph Fayrer spoke of the need there is of male nurses, and the suitability of employing them in various cases, with which we cordially agree, and contrasted the size and present working of the Association with that of six years ago. The audience oddly enough was composed largely of ladies.

### Notes and Queries.

To Correspondents.—1. Questions or answers may be written on post-cards. 2. Advertisements in disguise are inadmissible. 3. In answering a query please quote the number. 4. A private answer can only be sent in urgent cases, and then a stamped addressed envelope must be enclosed. 5. Every communication must be accompanied by the writer's full name and address, not necessarily for publication. 6. Correspondents are requested to help their fellow nurses by answering such queries as they can.

### Answers.

*Anxious.*—The Birmingham and Midland Counties' Training Institution for Nurses, Birmingham, is one, and the Queen's Hospital Nurses' Home, Birmingham, just started or about to start, is another.

*A Constant Reader.*—Anti-calcaire, or perhaps simply boiling your water before use, might bring about the result for you.

*Nurse Luke.*—The Matron of a cottage hospital should not have less than one month's annual holiday. She has seldom any difficulty in getting a substitute for that time, as many hospital Sisters are glad to get the experience, which can only be obtained by taking sole charge of an established hospital, whether it be a small or large one.

*S. H.*—We are constantly asked the question, but there is no institution, so far as we can learn, which makes a business of sending nurses abroad. There are sometimes advertisements in this journal asking for nurses to go abroad.

A nurse kindly writes to tell us of a very comfortable boarding house, which would be suitable for a nurse's holiday. The House of Rest, Burlington Place, Eastbourne, board and bed-room, with two or three beds in it, 7s. per week; board and separate bed-room, 12s. per week. Return fare for a month, 5s. All inquiries to be sent to *Miss Mason, House of Rest for Christian Workers, 10, Finchley Road, St. John's Wood.*

*Reader of* "The Hospital."—You must enter a hospital and go through the regular course of training to become a certificated nurse. You would doubtless find the subjects you mention of much help and interest in your nursing work, but without the practical and technical knowledge, which you can only acquire in a hospital, you cannot become a nurse of any sort.

### The Real Mrs. Skeffington.

When the Skeffingtons took Myrtlebank there was naturally a good deal of talk in the village. For one thing, nobody knew who they were or where they came from, and in Barrow-cum-Easter that sort of thing was not customary. On the contrary, its inhabitants had conscientiously and most thoroughly dissected each other's private history. The skeleton of each family in the place had been trotted out, and every bone counted over before the gruesome thing was consigned back to its cupboard-home. That duty being faithfully performed, there was never any more throwing stones, for the good, old-fashioned reason that glass houses will not really stand such usage; and things had settled down to the dead level in which the Skeffingtons found them when he and she took Myrtlebank on a short lease.

Myrtlebank was quite the largest house of the place, and not to say exactly in the village, being within a neighbouring plantation. It was a tumbledown building, picturesque enough with its mantle of ivy, but it had been a long time uninhabited—and looked like it.

The hopes raised by the advent of the new tenants were doomed to crumble away, however. The Skeffingtons had decided not to be sociable. This was a distinct shock, seeing that it had been some little effort for the community to decide that the unknowns should be taken up at all. To have one's overtures coldly ignored was astounding, and, for a short time, the village felt annihilated. However, Barrow-cum-Easter, with a shake reasserted itself, and then, after its customary fashion began prodding for the Skeffingtons' skeleton; at least, they were determined to have that. In the first place the explorers began to dig up antecedents; rather unyielding and unremunerative labour they found it. But, nothing daunted, that which was lacking was supplied by fertile imaginations, and, in time, people became so accustomed to the sound of their own inventions that they grew to believe them.

For instance, it got to be reported, vaguely at first, that Doctor Skeffington had been in the Navy—a few pretty stiff expressions, overheard, originated that belief. Afterwards it was discovered that the new arrival was a doctor, without any designing intentions of setting up a rival practice to Doctor Clay. Possibly, the extraction of this information might be set down to the credit of Widow Wigsby, a worthy individual who filled the niche of charwoman-in-chief to the village, as her mother had done before her; the post, like that of the hangman in foreign parts, being hereditary in Barrow-cum-Easter. This worthy was the only native admitted by the newcomers within their gates; in consequence, the acquaintance of Widow Wigsby was greatly sought after. Not that she was able to bring out from the mysterious household much information, whatever comestibles her black bag, that insignia of her office, exported.

"Which I'm not one to fetch and carry what the eye sees and the ear hears out of families that I do for; I don't hold with sich. But I do say of the good gentleman, the doctor, he's one that's seen a deal of the world, I make no doubt; and, if you ask me candid, I must say I can't abide 'im!" and the widow's head swayed mysteriously, mandarin fashion. "And as for his good lady," she continued, "I should say she've 'ad her troubles, pore thing. Not that I've ever seen her close; but she's quite the lady, can't so much as pick up her hankercher when it falls, and seems always that dazed like." A curious summary of a gentlewoman; but we don't all see through the experienced eyes of a charwoman. "Then ther's Miss; she's of another sort quite; she's that active and spry, and that ready to turn her 'and to anythink. I don't much hold with them kind of ladies!" And the widowed head was again tossed. Indeed, one might say this worthy punctuated her narrative with head-tossings.

"Who in the world is 'Miss'?" demanded the village.

"I dunno, I'm sure!" nearly sprung off the widow's lips, but she saved her reputation for information in time. "Well, if so be as she ain't own sister to the missus, my name ain't Martha Wigsby."

That was how it came to be an undoubted fact, for the future, that Mrs. Skeffington's sister formed one of the new family who had become the sole engrossing topic of conversation, no minutiæ being considered too trifling to add to the spicy dish of daily gossip concocted out of the Skeffingtons. To be sure, it was more than tiresome that they should turn out to be such recluses; and when Sunday after Sunday went by without their appearance in the little village church people's faces began to grow significantly long, and whispers went about, the mildest of which were that the strangers must be Papists or Unitarians or even Bryanites. But Doctor and Mrs. Skeffington were of none of these persuasions; in truth, they were, possibly, of no creed whatever, having never troubled their heads about such momentous questions.

"You must keep these village people at bay, Julia, from the first," the Doctor enjoined, a grave-looking little man, with dark, shifty eyes.

"Well, I don't know if that's quite the best tack for us to go on," was the deliberately spoken answer of the lady whom Widow Wigsby styled Miss. "Wouldn't it be wiser to let some of the natives in, and then there'd be no appearance of mystery when it takes place? Any mystery would be so damaging, James."

"Mystery or no, they're not coming inside these doors. Why they'd find you out in no time; and then where should we be?"

"It's a miserably dull life," grumbled Julia.

"Have patience, can't you! It won't be for long now," said the doctor, in a low tone.

"You really think so?" Julia's voice also sank. "Is she growing weaker?"

"Day by day!" was the brief response.

If the speaker meant the poor lady known to the village as the Missus, he was right. A painfully sad disease was making steady and sure progress, and this woman's earthly span was meted out plainly enough. Even the pure sweet country air brought no relief to the sufferer who was fading swiftly out of life. It was a dreary thing to be thus surely dying in the midst of the Midsummer gladness around; and the invalid, who was carried out, daily, in a chair and placed on the untidy overgrown lawn, lay gazing with wide, wistful eyes on the surrounding leafy glamour of nature.

"The pore dear's not long for this world!" sighed Widow Wigsby from afar, as she came and went to Myrtlebank, still being a necessary adjunct to the meagre establishment which consisted of an old cook who, singularly enough, was deaf and dumb.

"Which it's lonesome for me," observed the widow, with regard to the afflicted domestic; "we can never 'ave any sort of a chat; it's only play-actin' and signs between us."

*(To be continued.)*

### Appointment.

Melbourne Hospital.—Nurse Louisa Lapwood has been appointed Sister of the male medical wards, 16 and 17, in this hospital. Nurse Lapwood was trained for the Birmingham Nurses' Institute. She was afterwards charge nurse at West Bromwich Hospital, and then private nurse at the Nurses' Institute, Weston-super-Mare, there she remained until this present appointment.

Cumberland Infirmary, Carlisle.—In accepting Miss Allen's resignation on Wednesday, the 20th, the Committee of Management placed on record their appreciation of the great services rendered by her to this Infirmary in a time of special difficulty, and their entire satisfaction with her work. Miss Allen goes to Birmingham with many good wishes from her friends, and excellent testimonials from the medical staff of the Infirmary.

## Figures and Tables

**Figure f1:**